# Pediatric endoscopic pilonidal sinus treatment (PEPSiT): what we learned after a 3-year experience in the pediatric population

**DOI:** 10.1007/s13304-021-01094-4

**Published:** 2021-05-22

**Authors:** Ciro Esposito, Ernesto Montaruli, Giuseppe Autorino, Mario Mendoza-Sagaon, Maria Escolino

**Affiliations:** 1 Pediatric Surgery Unit, Federico II University of Naples, Naples, Italy; 2 Pediatric Surgery Unit, Ospedale Regionale Bellinzona e Valli, Bellinzona, Switzerland; 3 Division of Pediatric Surgery, “Federico II” University of Naples, Via Pansini 5, 80131 Naples, Italy

**Keywords:** PEPSiT, Fistuloscope, Laser, Dressing, Technique, Teenagers

## Abstract

This paper aimed to report a multi-institutional 3-year experience with pediatric endoscopic pilonidal sinus treatment (PEPSiT) and describe tips and tricks of the technique. We retrospectively reviewed all patients < 18 years, with primary or recurrent pilonidal sinus disease (PSD), undergoing PEPSiT in the period 2017–2020. All patients received pre-operative laser therapy, PEPSiT and post-operative dressing and laser therapy. Success rate, healing rate/time, post-operative management, short- and long-term outcome and patient satisfaction were assessed. A total of 152 patients (98 boys) were included. Median patient’s age was 17.1 years. Fifteen/152 patients (9.8%) presented a recurrent PSD. All patients resumed full daily activities 1 day after surgery. The post-operative course was painless in 100% of patients (median VAS pain score < 2/10). Patient satisfaction was excellent (median score 4.8). The median follow-up was 12.8 months (range 1–36). Complete healing in 8 weeks was achieved in 145/152 (95.4%) and the median healing time was 24.6 days (range 16–31). We reported post-operatively immediate Clavien grade 2 complications (3 oedema, 2 burns) in 5/152 (3.3%) and delayed Clavien grade 2 complications (3 granulomas, 8 wound infections) in 11/152 (7.2%). Disease recurrence occurred in 7/152 (4.6%), who were re-operated using PEPSiT. PEPSiT should be considered the standard of care for surgical treatment of PSD in children and teenagers. PEPSiT is technically easy, with short and painless post-operative course and low recurrence rate (4.6%). Standardized treatment protocol, correct patient enrollment and information, and intensive follow-up are key points for the success of the procedure.

**Supplementary Information:**

The online version contains supplementary material available at 10.1007/s13304-021-01094-4.

## Introduction

Pediatric endoscopic pilonidal sinus treatment (PEPSiT) represents the new frontier in the surgical management of pilonidal sinus disease (PSD) in the pediatric population. In fact, since its first report in the pediatric population in 2017 [1, 2], PEPSiT has dramatically revolutioned the surgical approach to pediatric patients with PSD. This technique provided a safe, effective and real minimally invasive care for both primary and recurrent PSD, that may be a socially invalidant condition especially in teenagers [3, 4]. The endoscopic treatment overcame all disadvantages reported by traditional open repair, such as the high recurrence rate up to 30%, the long and painful post-operative course and the long absence from school/work activities, causing a very bad psychological impact on the younger patients [5–7].

The main objective of the surgical management should be focused to treat not only the lesion but also all risk factors associated with disease recurrence. The most common risk factors for recurrence are represented by poor local hygiene and re-growth and re-accumulation of hair in the treatment area [8, 9]. To date, laser therapy represents the most effective method to achieve radical hair removal in the disease area [10]. A recent systematic literature review explained the importance of laser hair removal in patients treated for PSD, reporting a recurrence rate of 9% in patients undergoing laser therapy, 23.4% in those receiving razor shaving/cream depilation and 19.7% in those who had no hair removal after surgery for PSD [11]. These results confirmed that laser therapy was essential in the treatment of PSD.

For this reason, we recently standardized a multistep treatment protocol for PSD, consisting of a pre-operative phase using laser epilation therapy, an operative phase using the surgical technique PEPSiT and finally a post-operative phase including wound dressing using a specific oxygen-enriched oil-based gel and laser epilation therapy [12]. We reported excellent results using this multistep protocol for PSD, as we recently published [12, 13].

In these 3 years of experience with the technique, we experienced some tips, tricks and technical expedients to improve the technique, that we would like to share with the pediatric surgery community [14].

This paper reported a multi-institutional 3-year experience with PEPSiT with the aim to evaluate its effectiveness and safety in the pediatric population and describe the tips and tricks of the technique.

## Materials and methods

We retrospectively collected the data of all patients under 18 years of age, who received PEPSiT in two Pediatric Surgery Units over a 3-year period (2017–2020). Patients with recurrent PSD, following open repair or PEPSiT, were also included. Patients, who were older than 18 years at the time of surgery, were excluded.

All patients received the multi-step treatment protocol for PSD, that we have recently standardized [12–14]. This protocol included three steps, represented by pre-operative laser epilation therapy, surgical procedure using PEPSiT and post-operative wound management with specific dressing and laser therapy.

Follow-up included clinical appointments at 7, 14, 30, 60, 90, 180 days postoperatively and thereafter every 6 months. Data collection aimed to measure pre- and post-operative management, recovery time, post-operative pain management, healing rate/time, patient satisfaction and short- and long-term outcome.

The primary outcome of the study was to assess the success rate of surgery and the healing rate/time.

Surgical success was defined as the complete wound healing by 8 weeks postoperatively.

Secondary outcomes included recurrence rate, intra- and post-operative complications, post-operative pain and patient satisfaction. Post-operative complications were graded using Clavien classification system [15]. Post-operative pain was assessed using the visual analog scale (VAS). Patients scored their satisfaction level about post-operative course of PEPSiT using a five-items Likert-type scale (1–5), with 1 = very poor, 2 = poor, 3 = average, 4 = good, 5 = excellent.

The study received the appropriate Institute Review Board (IRB) approval at each participating center.

Written informed consent was obtained by participants to the study.

We described the following tips and tricks of PEPSiT technique.

### Pre-operative management

The laser epilation therapy was started before surgery with a minimum of two sessions every 4–6 weeks. The patients were also advised to do mechanical epilation of the intergluteal crease every week at home to avoid foreign body around the tips. The aim of pre-operative laser therapy was to remove the hair in the treatment area and prevent a rapid re-growth and re-accumulation of hair in the wound area postoperatively.

A Multi Variant Pulsed Light (MVPL) Laser was adopted in all cases. It was provided with a cooling system that made the treatment painless without the need to use local anesthesia, gel or any cooling methods.

The treatment area included the intergluteal sulcus and a 5-cm margin from the midline on each side (left/right) of the crease by 12 cm (cranial/caudal) from the apex to the anal opening.

Each treatment session lasted about 10 min with a median number of 100 spots per each session.

### Instrumentation

An adequate instrumentation was required to perform PEPSiT. The fistulectomy set, manufactured by KARL STORZ SE & Co. KG, Tuttlingen, Germany, included a 10 Fr fistuloscope, with 8° angled eyepiece, an outer diameter of 3.3 × 4.7 mm and a working length of 18 cm. A removable handle allowed to rotate the fistuloscope in different angulations and achieve a good ergonomics. The instruments included an endoscopic grasping forceps, rotating, with double action jaws, size 2 mm, length 30 cm, a 7 Fr monopolar electrode, and a fistula brush with insert of different outer diameter (4, 4.5 and 5 mm), for single use.

### Operative theatre setting

Before starting PEPSiT, the adequate setting of the operative theatre was crucial. Two monitors were placed, one at patient’s head and one at patient’s feet, alternatively adopted in accordance to the fistula’s cranial or caudal extension. The main surgeon was located at the patient’s right with the assistant on the contralateral side. The patient was placed in prone position, with the buttocks retracted with adhesive tape. The Trendelenburg angle of the operative table was increased at 10° and the patient was covered using waterproof sheets to avoid the flooding of the operating room during the procedure.

### Operative technique

**Fig. 1:**
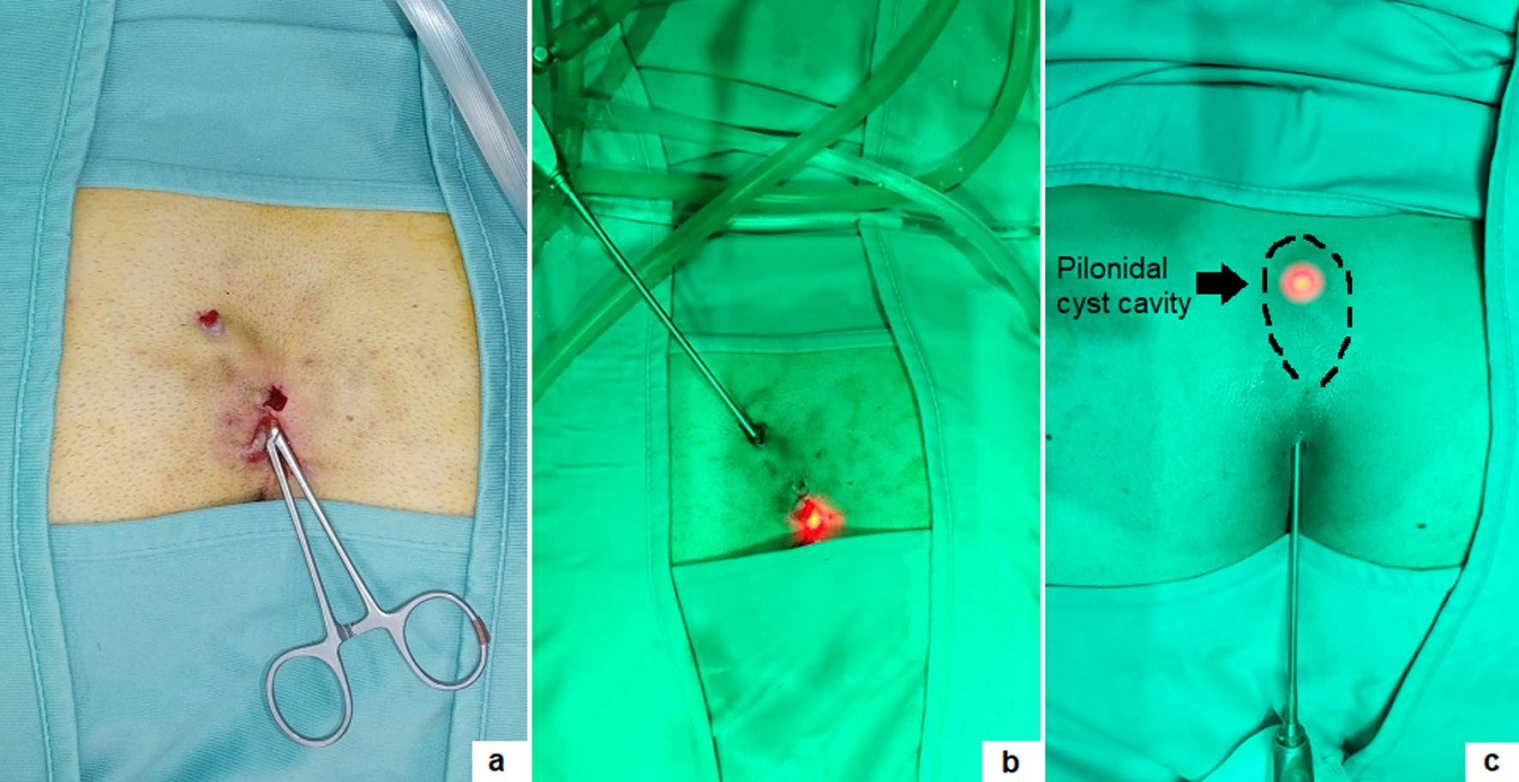
Communicating pilonidal cyst was reached introducing a clamp (**a**) or the fistuloscope through the external pit (**b**, **c**)

The standardized five-step operative technique, that we already described in previous papers [1, 12], was adopted in all patients (Video 1). We would report some tips and tricks emerged from our recent experience. If the external orifice is too narrow to allow the introduction of the fistuloscope, it can be widened using a spreading clamp or a metallic probe and then injecting saline using a syringe to enlarge the cavity. In presence of multiple pits, the lower one should be used to introduce the fistuloscope. It is very important to identify all secondary tracts or concomitant cyst. Pilonidal cyst is defined as an abnormal saclike structure in the skin, containing hair and skin debris, that is almost always located at the top of the cleft of the buttocks and may be or not in direct communication with the main fistula tract. In case of a pilonidal cyst not communicating with the main fistula tract, as depicted in Fig. 1, its wall may be opened using a spreading clamp introduced through the external pit or directly introducing the fistuloscope and its content may be evacuated by applying external hand compression. Finally, the inner aspect of the cyst cavity is treated with brushing and cauterization as a standard PSD.

**Fig. 2:**
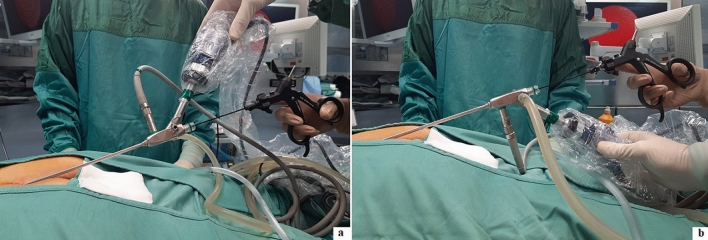
Fistuloscope can be adopted in normal position (**a**) or in upside down position (**b**)

All the hair present in the cavity should be removed. Since the fistuloscope has an 8° angled eyepiece, it may be difficult to reach the fistula roof. It may be useful during the procedure turn the fistuloscope upside down to check the fistula roof, remove all the present hair and coagulate the upper side of the cavity (Fig. 2).

Another important point was to debride the inner aspect of the fistula using the endobrush; however, we noted that when introducing the endobrush into a small cavity, the outer sheath completely occluded the working channel of the fistuloscope, limiting the passage of the irrigation solution and thus avoiding a good view of the operative field. In such cases, especially when the hair is numerous, the brush can be employed without direct vision to remove all the hairs and granulation tissue.

The last key point was to cauterize the inner aspect of the fistula all around. The monopolar coagulation was set at spray modality and we preferred to use a 0.54% mannitol/2.7% sorbitol irrigation solution, because it allowed to concentrate the energy just at the top of the instrument and avoid the risk of electric injury. It was important to respect the temperature of the liquid and provide a constant pressure by gravity without using any pump. It was very helpful just after surgery to push on the buttocks to allow the discharge of the introduced liquid and to perform a compressive dressing on the buttocks for at least 8–12 h postoperatively to reduce the risk of subcutaneous oedema. At the end of the procedure, it was very important to cauterize the external pits using monopolar coagulation at spray modality setting.

### Post-operative management

After hospital discharge, family members/caregivers were instructed to perform daily wound dressing. They applied topically a 2% eosin solution and an oxygen-enriched oil-based gel and covered the wound using a hyaluronic acid based wet gauze. It is very important to inject the product, introducing the tip of the syringe directly within the cavity. In this way, a higher concentration of the product was applied deeply in the wound area to increase the release of nascent oxygen and its beneficial effects on the wound healing [16, 17]. These dressings were repeated twice a day until completion of wound healing.

When the wound was healed, all patients received laser epilation sessions every 4–6 weeks. Completion of laser therapy was defined as no visible hair within an 8 cm by 12 cm zone around the initial PSD site. Furthermore, we advised the patients to keep the wound clean and dry, to wash the perineal region after each visit to the toilet and to accurately dry it after washing.

## Results

A total number of 152 patients (98 boys and 54 girls) received PEPSiT in all centers. Median patient’s age at surgery was 17.1 years (range 11–18) with just 48 patients < 16 years (31.5%). Comorbidity included obesity (*n* = 9), ulcerative colitis (*n* = 3), diabetes type 1 (*n* = 2), rheumatoid arthritis (*n* = 1), Down syndrome (*n* = 1). Fifteen out of 152 patients (9.8%) presented a recurrent PSD, following open repair (*n* = 8) and PEPSiT (*n* = 7). Two patients had received multiple open procedures (ranging from 3 to 6) in general surgery units before undergoing PEPSiT in our unit.

Most patients (129/152, 84.9%) had a pilonidal fistula with only 23/152 patients (15.1%) presenting a pilonidal cyst, that was not directly communicating with the main fistula tract. The median number of orifices was 2 (range 1–8). External pits were in the midline in most patients (144/152, 94.7%), whereas the pits were paramedian to the natal cleft in only 8/152 (5.3%). The cavity size was small (1–3 cm) in most cases (79/152, 52%), medium (3–5 cm) in 33/152 (22%) and large (> 5 cm) in 40/152 (26%). Most patients (123/152, 80.9%) had phenotypes dark (Fitzpatrick skin type III–IV) and significant hirsutism with a significant hair volume in the intergluteal crease.

Patients’ demographics/baseline is reported in Table 1.

**Table 1:** Patients’ demographics/baseline

Patients’ demographics/baseline	Value
Number of patients, *n*	152
Gender male, *n*	98
M:F	1.8:1
Median age, years (range)	17.1 (11–18)
Median weight, Kgs (range)	75.5 (58–105)
Comorbidity	
Obesity, *n* (%)	9 (5.9)
Ulcerative colitis, *n* (%)	3 (1.9)
Diabetes type 1, *n* (%)	2 (1.3)
Rheumatoid arthritis, *n* (%)	1 (0.6)
Down syndrome, *n* (%)	1 (0.6)
Recurrent PSD, *n* (%)	15 (9.8)
Clinical presentation	
Pilonidal fistula, *n* (%)	129 (84.9)
Pilonidal cyst, *n* (%)	23 (15.1)
Median number of orifices, *n* (range)	2 (1–8)
Midline pits, *n* (%)	144 (94.7)
Paramedian pits, *n* (%)	8 (5.3)
Cavity size	
Small (1–3 cm), *n* (%)	79 (52)
Medium (3–5 cm), *n* (%)	33 (22)
Large (> 5 cm), *n* (%)	40 (26)
Fitzpatrick skin type	
I, *n* (%)	0
II, *n* (%)	29 (19.1)
III, *n* (%)	54 (35.5)
IV, *n* (%)	69 (45.4)
V, *n* (%)	0
Hirsutism, *n* (%)	123 (80.9)

Surgical procedures were performed using saddle spinal block or loco-regional anesthesia in most cases (139/152, 91.4%). The surgical procedures were performed by two main surgeons in each center. No complications occurred intra-operatively. All patients resumed full daily activities 1 day after surgery. The post-operative course was painless in 100% of patients (median VAS pain score < 2/10). The median length of stay (LOS) was 8 h (range 6–24). An overnight hospitalization was required in some cases, according to the patient and family compliance. Patient satisfaction was excellent in all cases (median score 4.8).

**Fig. 3:**
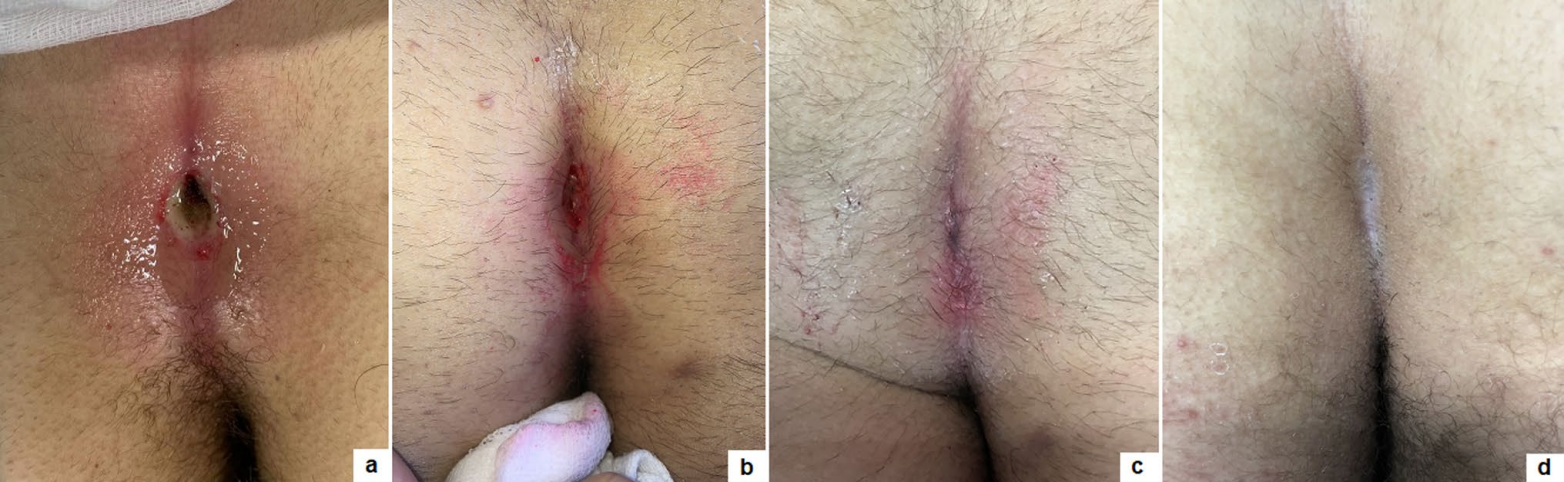
Wound healing outcome of primary PEPSiT at 1 (**a**), 7 (**b**), 14 (**c**), 21 (**d**) days postoperatively

The median follow-up length was 12.8 months (range 1–36). Complete healing in 8 weeks was achieved in 145/152 (95.4%) and the median healing time was 24.6 days (range 16–31) (Fig. 3). We reported immediate Clavien grade 2 post-operative complications (3 oedema, 2 fistuloscope burns) in 5/152 (3.3%) and delayed Clavien grade 2 post-operative complications (3 granulomas, 8 wound infections) in 11/152 (7.2%). Disease recurrence occurred in 7/152 (4.6%) patients, who were re-operated using PEPSiT with no further recurrence. Laser epilation was well tolerated in all patients. No laser-related complications, such as thermal injuries or skin pigmentation changes, occurred.

Outcomes of our PEPSiT series are reported in Table 2.

**Table 2:** Outcomes of our PEPSiT series

Outcome parameter	Value
Anesthesia type	
Saddle spinal block, *n* (%)	83 (54.6)
Loco-regional anesthesia, *n* (%)	56 (36.8)
General anesthesia, *n* (%)	13 (8.6)
Median operative time, minutes (range)	28 (18–65)
Intra-operative complications, *n* (%)	0
Median VAS pain score (12 h after surgery)	< 2/10
Median length of stay, hours (range)	8 (6–24)
Median time to full daily activities, days (range)	1 (0–1)
Median follow-up length, months (range)	12.8 (1–36)
Complete healing in 8 weeks, *n* (%)	145 (95.4)
Median healing time, days (range)	24.6 (16–31)
Post-operative complications	
* Clavien grade 2, overall n (%)*	16 (10.5)
Oedema, *n* (%)	3 (1.9)
Fistuloscope burn, *n* (%)	2 (1.3)
Wound infection, *n* (%)	8 (5.3)
Granuloma, *n* (%)	3 (1.9)
Recurrence, *n* (%)	7 (4.6%)
Median patients’ satisfaction score (1–5), *n* (range)	4.8 (4.2–5)

## Discussion

Based upon our 3-year experience, we outline some key points for the success of PEPSiT protocol.

**Fig. 4:**
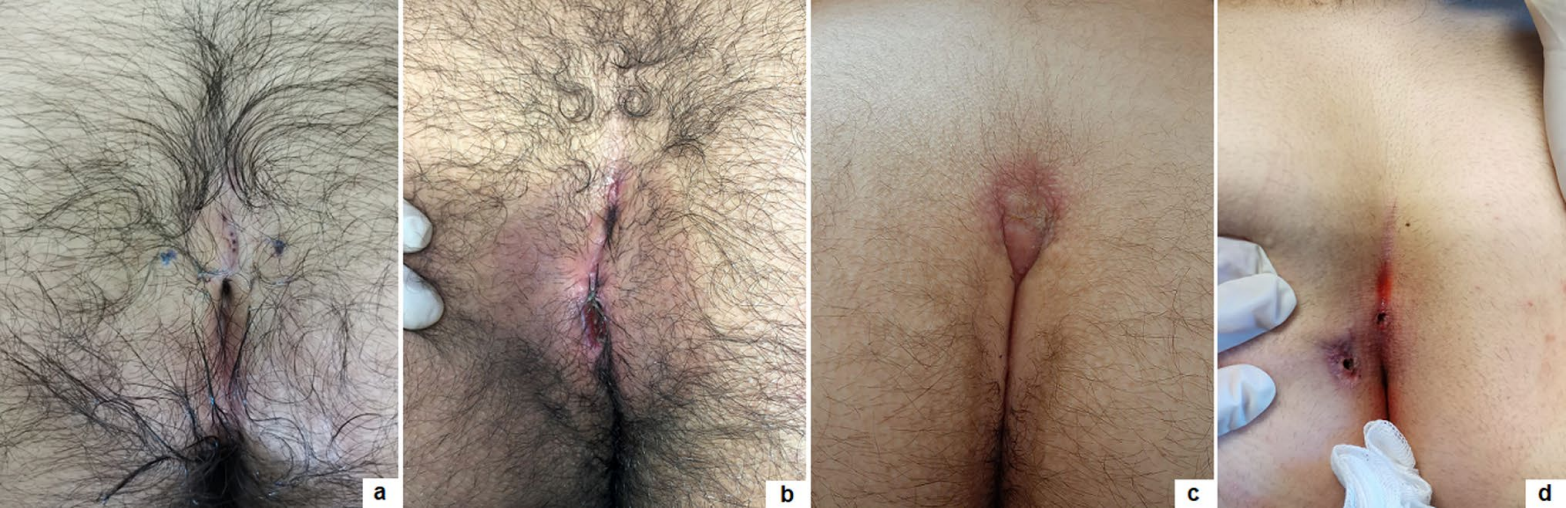
Clinical presentations of primary PSD: multiple non secreting median pits (**a**); multiple secreting median pits (**b**); pilonidal abscess (**c**); median and paramedian pits (**d**)

**Fig. 5:**
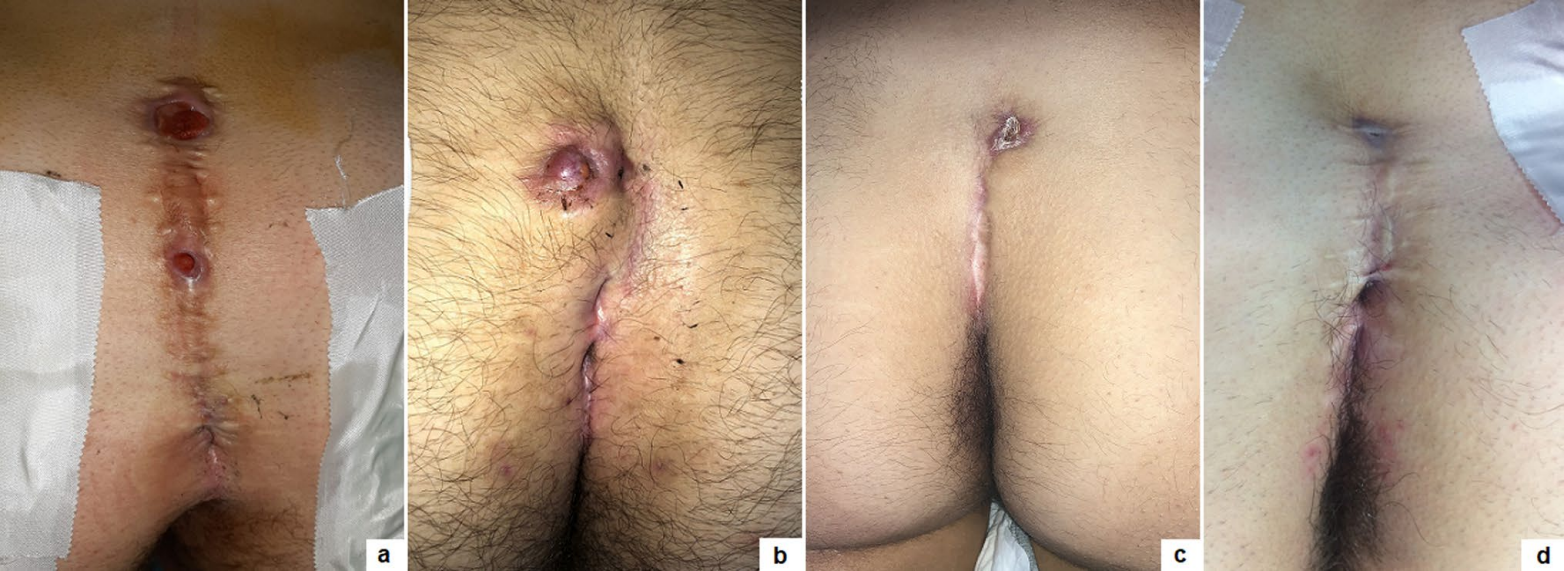
Clinical presentations of recurrent PSD: multiple median granulomas (**a**); multiple secreting median pits and paramedian granuloma (**b**); single non secreting paramedian pit (**c**); multiple non secreting median pits

**Fig. 6:**
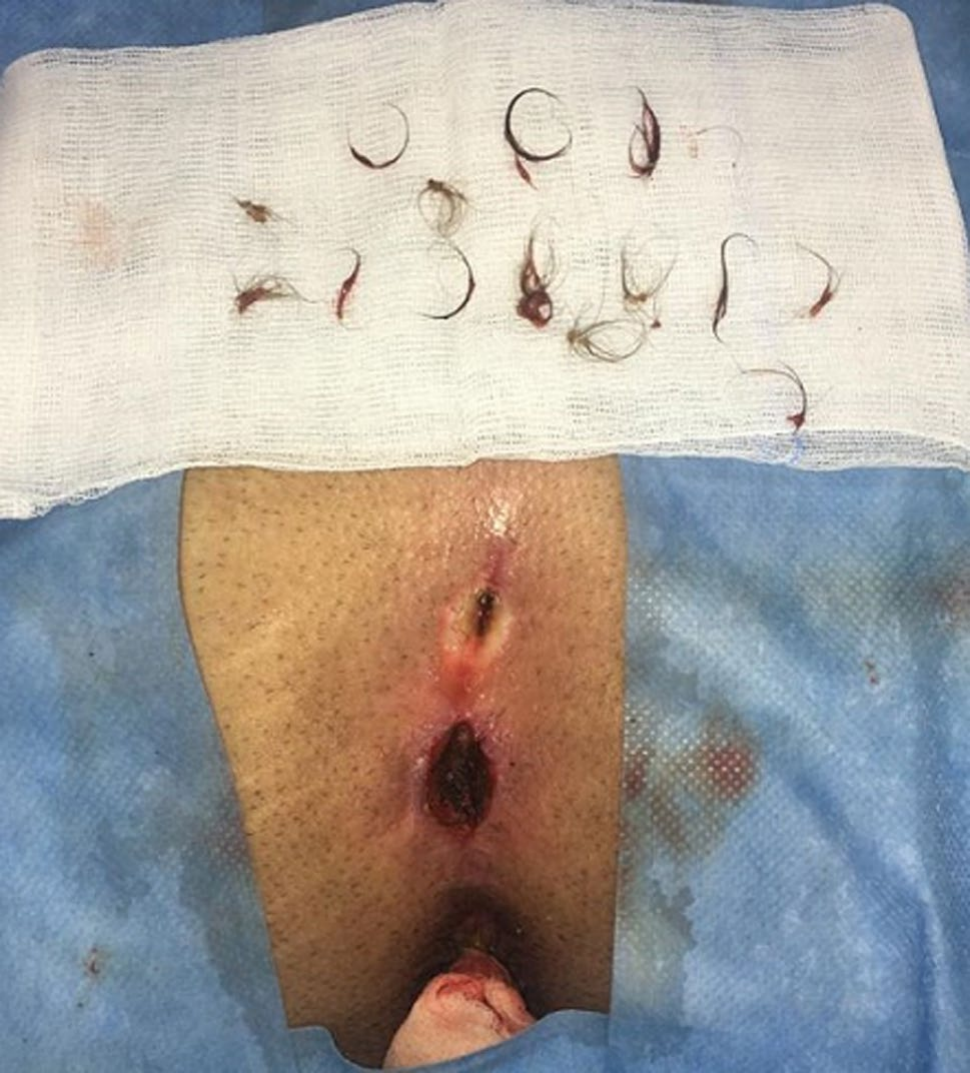
Hair removed from the cavity at the end of procedure

**Fig. 7:**
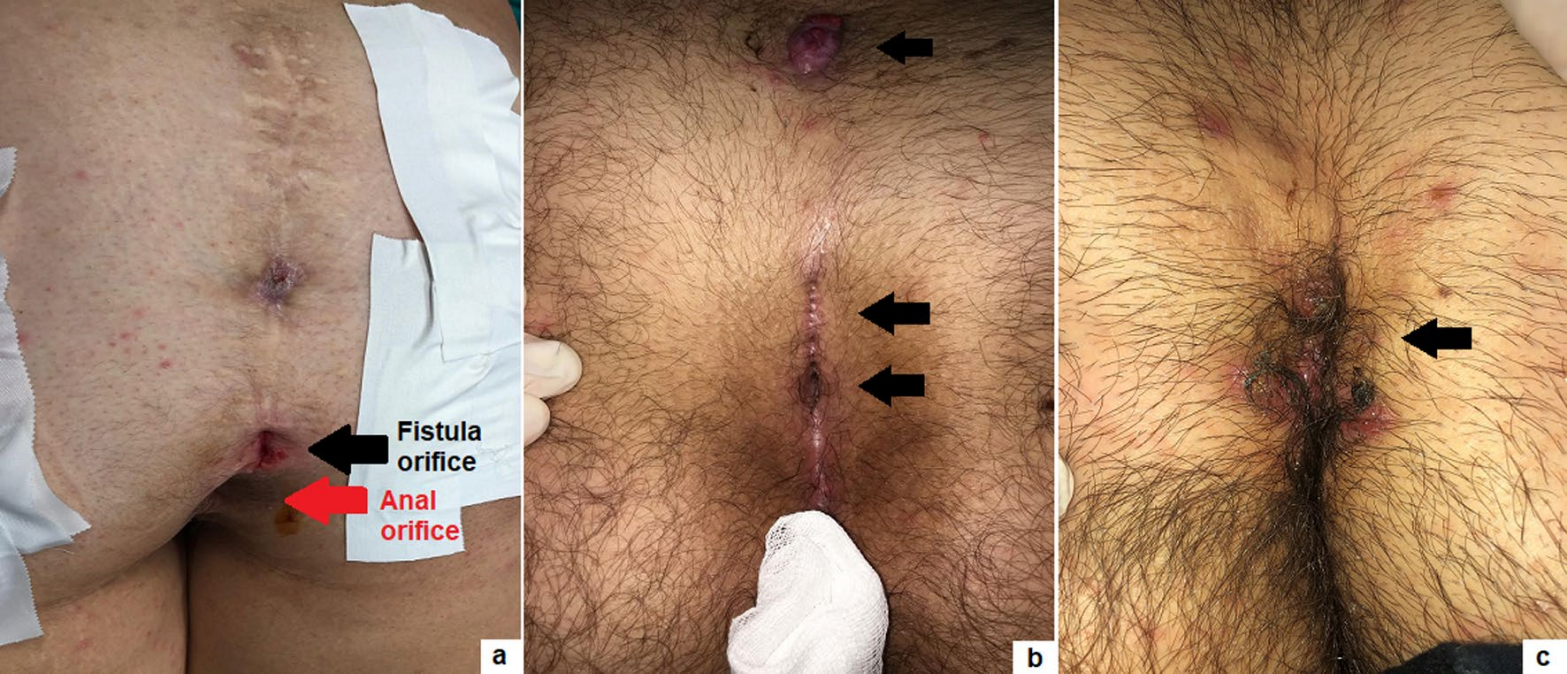
Terrible three: too low pit (*black arrow*) close to the anal orifice (*red arrow*) (**a**); too many (**b**) pits (*black arrows*); too hairy (**c**) around the PSD site (*black arrow*)

1. Know and inform your patient: the success rate of PEPSiT depends on the patient compliance and adherence. It is very crucial to renovate some information to the patient before surgery, especially improving personal hygiene to reduce the risk of infection. It is also correct to inform the patient that the follow-up could be challenging and long.
2. Prepare your patient: the hair removal protocol should be started as soon as possible. The laser epilation therapy should be performed before surgery with a minimum of two sessions every 4–6 weeks. This is a teamwork in our units and it is important to respect the physiological gap between sessions to achieve the best results with the laser therapy. In fact, it seems that laser energy works well just during the anagen and catagen phase of the hair growth cycle [18, 19]. You must also remember that energy is like heat and like burns when you perform laser therapy. It is also important to advise the patient to do mechanical epilation of the intergluteal crease every week at home to avoid foreign body around the tips.
3. Correct patient enrollment: fast but not furious. It is crucial to study properly the patient and establish if the patient has only pits, a fistula, a recent abscess, a pilonidal cyst and if it is really a PSD (Figs. 4, 5). In fact, some other clinical conditions, such as a spinal dysraphism, may simulate a PSD [20]. In such cases, imaging examinations, such as ultrasound (US) or magnetic resonance imaging (MRI), may be helpful to clarify the diagnosis. The presence of a pilonidal cyst, even if wide in size, does not constitute a limitation or a contraindication to PEPSiT. In 15.1% of patients of our series, we found a pilonidal cyst that was not directly communicating with the main fistula tract. In such cases, it is crucial to open the cyst wall using a spreading clamp introduced through the external pit or directly introducing the fistuloscope and evacuate its content by applying external hand compression. Finally, the inner aspect of the cyst should be treated with brushing and cauterization as a standard PSD.
4. Surgical conditions: technically, the fistuloscopy is like a cystoscopy. First of all, it is important to respect the “water” conditions and choose the correct type of irrigation solution; personally, we use a 0.54% mannitol/2.7% sorbitol solution, because it permits to concentrate the energy just at the top of the instrument and avoid the risk of electric injury. It is important to respect the temperature of the liquid and provide a constant pressure by gravity without using any pump. The risk of subcutaneous oedema exists but it is important to push on the buttocks just after surgery to allow the discharge of the introduced liquid and to perform a compressive dressing on the buttocks for at least 8–12 h postoperatively to reduce the subcutaneous oedema formation. It is also important to avoid flooding in the operative theatre and protect yourself and the patient. Adopting waterproof sheets to cover the patient, using a slight (10°) Trendelenburg angle of the operative table and using a continuous suction system are some tricks to avoid flooding in the operating room.
5. Recognize the barrow: it is important to remove all granulation tissue, recognize the nest of hair and remove fibrin and all hair from the wound (Fig. 6). Recurrent PSD is a very good indication for PEPSiT. In some patients with recurrent PSD, especially females, we typically found no hair but also fibrin and epithelial tissue; in such cases, it is important to coagulate the inner aspect of the cavity all around to ablate the epithelial secreting line responsible for PSD recurrence. In other cases, we found variable amount of hair that had grown back after the first surgery and we removed it. It is also important to be careful with the skin, because an excessive cauterization may transform normal tissue in necrotic tissue.
6. Standardize the operative technique: adequate instrumentation, proper setting of the operative theatre and experience of the surgical team are key factors for the success of the procedure [12].
7. Recognize the terrible **t**hree (too low, too many, too hairy): too low is when the external pit is too proximal to the anus; too many is when there are more than two pits and too hairy is when there is a huge number of hair in particular in hirsute patients (Fig. 7). The presence of these three conditions may sometimes complicate the post-operative period and increase the risk of disease recurrence.
8. Ensure an intensive follow-up and be realistic and not too optimistic during the follow-up. It is important to remember that behind a hole there is a cave, because sometimes you have just a little plug of fibrin that occludes the pit and the cavity is not completely discovered; in such cases, it is important to remove the plug to identify and clean the cavity. The intensive follow-up is composed by regular laser epilation, serial wound dressing until complete re-epithelization and regular specialist follow-up. If you work well, 3 days after the procedure, patients may return to normal physical activity, play football, volleyball or ski.

Now, you are ready for a PEPSiT. It is important to prepare your patient, be peremptory on the pre- and post-operative course, and remove all visible hair. You must be careful with the enrollment, prevent flooding, oedema and protect the patient and yourself during the procedure, standardize your technique and be realistic and not too optimistic during the follow-up. Finally, you must remember the terrible three and inform the patient correctly that in such case the healing time will be longer than the others.

In conclusion, based upon our 3-year experience, we believe that PEPSiT should be considered the standard of care for surgical treatment of PSD in children and teenagers. PEPSiT is technically easy, with a short and painless post-operative course and a low recurrence rate (4.6%). However, standardized treatment protocol, correct patient enrollment and information, standardized operative technique and intensive follow-up are key points for the success of the procedure. Personally, we think that open excision with primary or secondary intention closure is the past and PEPSiT should be considered the present and the future for surgical management of children and teenagers with PSD.

## Availability of data and material (data transparency)

All data generated or analysed during this study are included in this published article [and its supplementary information files].

## Supplementary Information

Below is the link to the electronic supplementary material.

**Video 1**: PEPSiT operative technique (MP4 202191 KB)
